# Rates of Medical Student Placement Into Graduate Medical Education by Sex, Race and Ethnicity, and Socioeconomic Status, 2018-2021

**DOI:** 10.1001/jamanetworkopen.2022.29243

**Published:** 2022-08-26

**Authors:** Mytien Nguyen, Sarwat I. Chaudhry, Mayur M. Desai, Alexandra M. Hajduk, William A. McDade, Tonya L. Fancher, Dowin Boatright

**Affiliations:** 1 Yale School of Medicine, New Haven, Connecticut; 2 Section of General Internal Medicine, Department of Medicine, Yale School of Medicine, New Haven, Connecticut; 3 Department of Chronic Disease Epidemiology, Yale School of Public Health, New Haven, Connecticut; 4 Section of Geriatrics, Department of Medicine, Yale School of Medicine, New Haven, Connecticut; 5 Accrediation Council for Graduate Medical Education, Chicago, Illinois; 6 Division of General Internal Medicine, University of California, Davis, School of Medicine. Sacramento; 7 Department of Emergency Medicine, New York University Grossman School of Medicine, New York

## Abstract

This cohort study investigates whether different rates of medical student placement into graduate medical education exist by sex, race and ethnicity, and socioeconomic status from 2018 to 2021.

## Introduction

Between 2005 and 2015, American Indian or Alaska Native, Asian, Black, and Hispanic medical graduates were less likely to transition into graduate medical education (GME) compared with their White peers.^1^ Whether this disparity in GME placement persists across racial and ethnic or other marginalized identities remains unknown. In this study, we examine the likelihood of GME placement by race and ethnicity, sex, and socioeconomic status.

## Methods

We conducted a retrospective cohort study of 2014 to 2015 and 2015 to 2016 medical school matriculants who applied to residency from 2018 to 2021 using the Association of American Medical Colleges GME Track Resident Survey data. Sex and racial and ethnic identity were self-reported, and included: American Indian or Alaska Native, Asian, Black or African American, Hawaiian Native or Pacific Islander, Hispanic, Multiracial, and Unknown or Other. Underrepresented in medicine (URIM) students are students who identified as American Indian or Alaska Native, Black or African American, Hispanic, or Hawaiian Native or Pacific Islander alone or in combination with other racial and ethnic identities. Students with low income were defined as recipients of state and/or federal financial assistance or whose parental income was within the lowest 2 quintiles of households.

We compared placement rates across sex, race and ethnicity, and income using χ^2^ test, with 2-sided *P* < .05 indicating significance. Multivariate logistic regression was used to model the associations of intersectional sex, race and ethnicity, and low-income identities with GME placement, adjusting for US Medical Licensing Exam (USMLE) Step 2 scores. This study followed the STROBE reporting guideline and was deemed exempt by the Yale institutional review board. Statistical analyses were performed using Stata version 16.1 (StataCorp).

## Results

Among 37 485 students who applied to residency, 6387 (17.0%) identified as URIM, 18 193 (48.5%) were female, and 8423 (23.9%) had low income; 1741 (4.6%) were not placed in GME (Table). Black or African American and Hispanic male students, and American Indian or Alaska Native and Hawaiian Native or Pacific Islander female students had the highest rates of unsuccessful placement (Figure, A). In our adjusted model with sex, race and ethnic identities, and USMLE Step 2 scores, Black or African American male students (adjusted odds ratio [aOR], 1.78; 95% CI, 1.40-2.28), Hispanic male students (aOR, 1.53; 95% CI, 1.21-1.95), Black or African female students (aOR, 1.34; 95% CI, 1.06-1.69), and Hispanic female students (aOR, 1.37; 95% CI, 1.06-1.77) were more likely to not place in GME compared with White male students (Figure, B).

**Table.** Characteristics of Medical School Matriculants, 2014 to 2015 and 2015 to 2016, and Placement Into Graduate Medical Education

Characteristic	Matriculants, No. (%)		*P* value
	Total (N = 37 485)	Unplaced in GME (n = 1741)	
Sex			
Male	19 292 (51.5)	1002 (5.1)	<.001
Female	18 193 (48.5)	739 (4.1)	
Race and ethnicity^a^			
AIAN/HNPI	133 (0.4)	9 (6.7)	<.001
Asian	7337 (19.6)	330 (4.4)	
Black or African American	2344 (6.3)	182 (7.7)	
Hispanic	2311 (6.2)	165 (7.1)	
Multiracial	2243 (6.0)	114 (5.0)	
White	19 733 (52.6)	728 (3.6)	
Unknown or Other	3384 (9.0)	213 (6.2)	
Low income			
No	26 782 (71.4)	1161 (4.3)	<.001
Yes	8423 (22.5)	461 (5.4)	
Missing	2280 (6.1)	119 (5.2)	

Abbreviations: AIAN/HNPI, American Indian or Alaska Native and Hawaiian Native or Pacific Islander; GME, Graduate Medical Education, from the GME Track Resident Survey.

^a^ Racial and ethnic identity alone or with other. Students who reported more than 1 racial and ethnic identity were categorized as multiracial. AIAN and HNPI racial and ethnic identity were collected as distinct groups. Due to small sample size, we have aggregated these 2 racial and ethnic groups.

**Figure.**
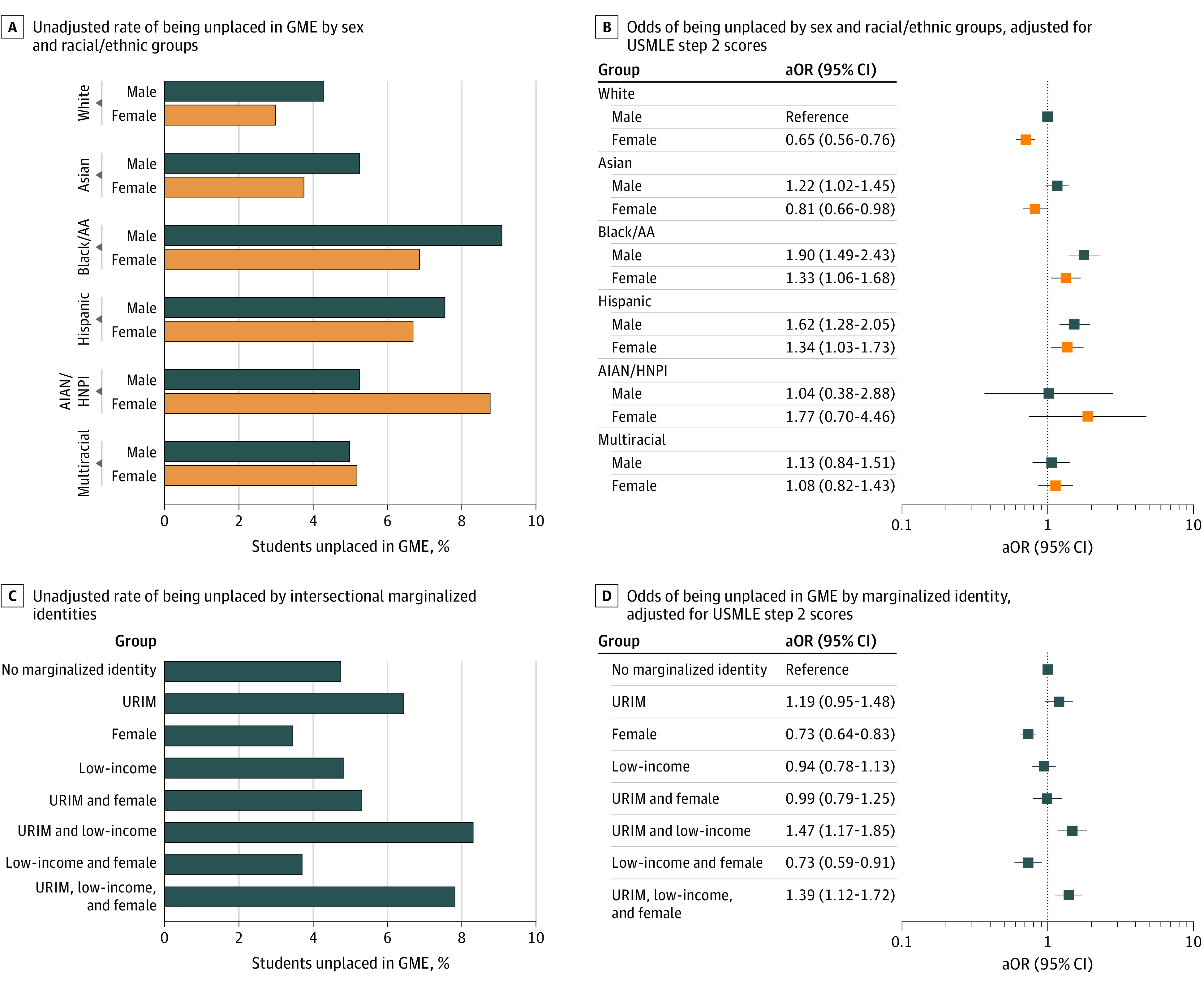
Rates and Adjusted Odd Ratios (aORs) of Being Unplaced in Graduate Medical Education (GME) AIAN/HNPI indicates American Indian or Alaska Native and Hawaiian Native or Pacific Islander; Black/AA, Black or African American; LI, low income; URIM, underrepresented in medicine (includes students who self-identify as Black or African American, Hispanic, American Indian or Alaska Native, Hawaiian Native or Pacific Islander alone or in conjunction with other ethnoracial identity); USMLE, US Medical Licensing Examination.

Multivariate logistic regression of sex–racial and ethnic identity with income showed that both low– and non–low-income URIM male students and low-income URIM female students had the highest rates of unsuccessful placement (Figure, C). In our fully adjusted model with USMLE Step 2 scores, low-income URIM male students (aOR, 1.47; 95% CI, 1.17-1.85) and female students (aOR, 1.39; 95% CI, 1.12-1.72) had the highest odds of not placing into GME compared with non–low-income, non-URIM male students (Figure, D).

## Discussion

We found that American Indian or Alaska Native, Black, Hawaiian Native or Pacific Islander, and Hispanic students had the highest unsuccessful GME placement rates, particularly URIM men and low-income URIM students. Low-income status seemed to only influence placement rates for URIM students.

These findings suggest that there may be potential bias in the GME placement process against Asian men and URIM students, especially URIM students with low income. Structural components of residency application, such as honor society membership, have shown class and racial and ethnic disparities^2,3^ which may limit hiring opportunities for low-income URIM students. The racial and ethnic disparity in GME placement rates is consistent with prior research from the business sector, where applicants from minoritized racial and ethnic groups are less likely than White applicants to receive a call back from potential employers despite similar qualifications.^4^

Our study is limited in that GME placement rates may vary across disaggregated racial and ethnic groups and low numbers of American Indian, Alaska Native, Hawaiian Native, and Pacific Islander students, as well as environmental factors (eg, medical school ranking). Examining placement rates across school characteristics, disaggregated racial and ethnic identities, and other marginalized identities (eg, disability status), is critical to understand disparities in GME placement.

Although most students place into GME after repeated attempts,^1^ the lower initial placement rate for URIM students may represent a previously undescribed minority tax requiring some students to prolong their time to independent practice, limiting their lifetime earnings potential, and restricting physician workforce diversity. Along with prior studies,^5,6^ this study suggests that URIM students with low income, and Black men in particular, experience attrition at multiple points during training. Potential interventions could include linking equity metrics, such as GME placement, to undergraduate and graduate medical education to establish greater transparency and accountability for outcomes concerning workforce diversity, equity, and inclusion.
